# Does Vancomycin as the First-Choice Therapy for Antibiotic Prophylaxis Increase the Risk of Surgical Site Infections Following Spine Surgery?

**DOI:** 10.3390/antibiotics14100996

**Published:** 2025-10-05

**Authors:** Vojislav Bogosavljevic, Dusan Spasic, Lidija Stanic, Marija Kukuric, Milica Bajcetic

**Affiliations:** 1 Clinic of Neurosurgery, University Clinical Center of Serbia, 11000 Belgrade, Serbia; vojislav.bogosavljevic@med.bg.ac.rs (V.B.); dr.lidija.stanic@kcs.ac.rs (L.S.); 2 Department of Pharmacology, Clinical Pharmacology and Toxicology, Faculty of Medicine, University of Belgrade, 11000 Belgrade, Serbia; dusan.spasic.dr@med.bg.ac.rs; 3 Department of Emergency Radiology, University Clinical Center of Serbia, 11000 Belgrade, Serbia; marija.kukuric@kcs.ac.rs

**Keywords:** surgical site infections (SSIs), spinal surgery, antibiotic prophylaxis, vancomycin, cefazolin, antimicrobial resistance (AMR)

## Abstract

Surgical site infections (SSIs) remain a significant complication in spine surgery, especially in instrumented procedures with long operative times. Although guidelines recommend cefazolin as the first-line agent due to its efficacy against Staphylococcus aureus, predictable pharmacokinetics, and safety, its real-world practice is highly variable, with inappropriate and prolonged regimens reported across Europe. Vancomycin is often used as the first choice of therapy empirically and without screening, exposing patients to risks such as delayed infusion, nephrotoxicity, and the emergence of vancomycin-resistant enterococci (VRE).This review assesses the present function of vancomycin in relation to cefazolin for spinal prophylaxis and examines wider trends in the misuse of surgical antibiotic prophylaxis, which were identified through PubMed and Scopus searches. Evidence from randomized and prospective studies consistently supports cefazolin as the preferred prophylactic agent in clean spinal surgery. Observational data suggest that adjunctive or topical vancomycin may reduce infection rates in selected high-risk or revision cases, though the results are inconsistent and frequently limited by retrospective designs and heterogeneous outcome reporting. Importantly, the most rigorous randomized controlled trial found no benefit of intrawound vancomycin over the placebo. A small number of available investigations in vancomycin use with major design limitations have resulted in no significant VRE emergency. Unexpectedly, widespread use of vancomycin was followed by a notable transition toward Gram-negative and opportunistic organisms. In summary, vancomycin may only be considered in patients with documented MRSA colonization, β-lactam allergy, or selected revision procedures, but its widespread empirical use as a first-choice therapy is not supported.

## 1. Introduction

Antimicrobial resistance (AMR) represents a significant challenge to modern medicine, threatening the effectiveness of treatments and the safety of procedures throughout the healthcare system. *The Lancet*’s landmark report highlighted that bacterial AMR was directly associated with more than 1.14 million deaths worldwide in 2021, and played a role in nearly 6 million fatalities overall [1]. This escalating crisis impacts a wide array of areas, jeopardizing the safety of standard interventions that rely on antimicrobial protection, such as surgical procedures. Projections indicate that, by 2050, antimicrobial resistance could lead to approximately 10 million deaths each year, particularly affecting surgical and critical care specialties the most severely [1,2].

The irrational use of antibiotics stands as one of the key factors contributing to AMR [1]. Globally, the improper management of infections is frequently highlighted; however, the preventive use of antibiotics, particularly when misapplied or excessively utilized, is also a crucial factor to consider. Surgical antibiotic prophylaxis (SAP) is widely recognized as a standardized antimicrobial strategy in clinical medicine; however, it is often applied with various inconsistencies. Recent point prevalence studies carried out in Europe revealed a significant variation in SAP between countries, ranging from 6.6% to 22.9% of all prescribed antimicrobials. In most countries, the majority of surgical patients were administered prophylaxis that was either poorly timed or excessive, which consequently heightened selective pressure on microbial flora and contributed to the evolution of resistance. The percentage of SAP prescribed outside of recommendation bounds was correlated with the composite index of antimicrobial resistance in hospital-acquired infections (HAIs) at country level [2,3].

In particular, spine surgery poses distinct challenges. The regular use of implants, the extended surgical times, and the complex anatomical factors increase the likelihood of surgical site infection (SSI). In an effort to prevent infection, antibiotics are commonly given; nonetheless, the increasing use of broad-spectrum agents like vancomycin—frequently prescribed without verified methicillin-resistant *Staphylococcus aureus* (MRSA) colonization—has generated considerable discussion. Importantly, the guidelines from the North American Spine Society (NASS) highlight that there is no conclusive evidence supporting the superiority of one prophylactic agent over others in standard spinal procedures. They advocate for making decisions based on individual patient risk factors and colonization status [4,5]. Nonetheless, vancomycin is commonly administered in approximately 30–40% of spinal surgeries at certain centers, even when high-risk indicators are not present [4].

This review examines the function of vancomycin in surgical prophylaxis, specifically in the context of spine surgery. We investigate the mechanisms and epidemiology of spinal SSIs, current prophylactic protocols, the pharmacological rationale for vancomycin use, and the clinical evidence concerning its outcomes and risks. Particular focus is directed towards the intriguing possibility that prophylactic vancomycin could potentially worsen the complications that it is intended to mitigate. This encompasses the identification of resistant Gram-negative organisms, as indicated by recent longitudinal studies [6]. This analysis seeks to elucidate the role of vancomycin in modern prophylactic protocols and underscore the importance of evidence-based antibiotic stewardship in spinal surgery.

## 2. Antibiotic Prophylaxis in Spine Surgery: Indications, Agents, and Guideline Recommendations

Antibiotic prophylaxis plays a vital role in perioperative care during spinal surgery, as managing surgical site infections (SSIs) is essential given the intricate nature of the procedures and the common use of implants. With the rise in spinal surgery volumes and the increasing complexity of cases, characterized by longer operative times, revision procedures, and the presence of aging or comorbid patients, the urgent need for evidence-based infection prevention is at an all-time high. While SSIs occur infrequently, their effects on clinical outcomes are significant, frequently requiring additional surgeries, removal of implants, and longer hospital admissions, and possibly resulting in lasting disability or functional impairment [7,8].

The incidence of SSIs in spinal surgery has been reported to vary between 0.7% and 12%, influenced by factors including instrumentation, operative duration, comorbidity burden, and surgical approach [8,9,10]. Instrumented thoracolumbar fusions exhibit heightened vulnerability owing to the expanded surface area of the hardware, which serves as a focal point for microbial colonization. The primary organisms associated with these infections are Gram-positive cocci, particularly *Staphylococcus aureus* (including both MSSA and MRSA) and coagulase negative staphylococci (CoNS). These organisms are recognized for their capacity to adhere to implant surfaces and establish protective biofilms [10,11]. Another important concern is the emergence of vancomycin-resistant enterococci (VRE), which has become a notable nosocomial threat in facilities with elevated vancomycin usage. VRE presents a unique and growing challenge in surgical wards and intensive care units. *Enterococci* are robust organisms that can endure on surfaces, linens, and medical equipment for prolonged durations. The presence of the vanA and vanB operons confers resistance to glycopeptides like vancomycin and promotes horizontal gene transfer within Gram-positive species. Following colonization, especially in high-risk settings like neurosurgical units, they can endure for extended periods, potentially facilitating transmission among at-risk patient groups. The presence of implanted hardware in invasive procedures adds complexity to the management of VRE infections, leading to extended hospital stays and the need for alternative treatment options.

Global and specialty specific recommendations consistently support the implementation of perioperative intravenous prophylaxis in clean spinal surgeries. The CDC and North American Spine Society (NASS) advocate for the administration of agents that are effective against likely pathogens within 60 min of incision [3,4,9]. Cefazolin continues to be the preferred first-line agent, valued for its consistent effectiveness against Gram-positive organisms, affordability, and established safety profile. Cefazolin not only exhibits a broad antimicrobial spectrum, but also has pharmacokinetic properties that render it particularly advantageous for spine surgery. Pharmacodynamic data indicates that cefazolin quickly distributes into vertebral bone, attaining concentrations that exceed the minimum inhibitory concentration (MIC) for *S. aureus* within 30–60 min of infusion [12]. A study indicated that therapeutic levels remained in cancellous bone tissue for as long as three hours after administering a single 2 g dose, highlighting the necessity for timely redosing in procedures that extend beyond this timeframe [12].

Standard dosing consists of administering 2 g IV within one hour before incision, with an increase to 3 g for patients weighing over 120 kg. Redosing during the intraoperative period is recommended after a duration of 4 h or in cases of blood loss exceeding 1.5 L [3,13]. Even with these clearly defined guidelines, the actual application in practice varies significantly. For instance, findings from a national point prevalence survey in Serbia indicated that cefazolin was utilized in only 18.4% of spinal surgeries, whereas ceftriaxone, a broad-spectrum agent not advised for clean surgical procedures, was employed in 21.3% [14]. Furthermore, prophylaxis frequently lasted beyond 48 h, with 71.4% of surgical patients undergoing SAP for over one day [14]. The observed deviations are devoid of clinical justification and further exacerbate the issues of antimicrobial resistance and the heightened risk of *Clostridioides difficile*.

Vancomycin is advised for individuals who have beta-lactam allergies or have been confirmed to be colonized with MRSA. Nonetheless, its wider application in primary cases without screening continues to be a subject of debate [10,15]. Vancomycin should be administered gradually over a period of at least 60 min to prevent infusion-related reactions like red man syndrome, and to achieve appropriate serum and tissue levels. In contrast to cefazolin, the penetration of vancomycin into spinal bone tissue is both delayed and variable, potentially undermining its efficacy during the critical initial contamination window of surgery [12]. If started too late or administered too quickly, inadequate bone levels at the time of incision may occur, especially in shorter procedures.

Dual-agent prophylaxis (cefazolin + vancomycin) has become increasingly recognized in high-risk populations, especially in the context of revision surgeries and multilevel instrumentations. This practice is supported by evidence showing a decrease in deep SSIs among certain subgroups; however, it also reveals significant concerns about nephrotoxicity, logistical challenges, and environmental implications [4,6]. The endorsement of this strategy varies among the guidelines. Some hospital-level protocols endorse combination prophylaxis in MRSA endemic environments, while others advise caution regarding its broad implementation, citing insufficient overall evidence and worries about the selection pressure for vancomycin-resistant enterococci (VRE) [4,16].

Occasionally, alternative agents like clindamycin and teicoplanin are utilized when both cefazolin and vancomycin are not suitable. Clindamycin exhibits bacteriostatic properties and demonstrates reduced efficacy in high-inoculum infections commonly associated with spinal hardware cases. Teicoplanin, in contrast, possesses a similar Gram-positive spectrum as vancomycin but exhibits a more advantageous safety profile and extended half-life, facilitating more adaptable perioperative dosing [4,15]. Certain pharmacokinetic studies indicate enhanced bone penetration and a lower likelihood of nephrotoxicity associated with teicoplanin, yet there is a scarcity of comparative trials specifically in the context of spinal prophylaxis.

Throughout various guidelines, three core principles of SAP are consistently highlighted: (1) selecting agents aimed at anticipated flora; (2) administering treatment within 60 min of incision; and (3) discontinuing within 24 h after clean procedures [3,4,9]. However, institutional audits consistently uncover deficiencies in adherence to regulations. Infusion timing delays, extended postoperative dosing, and an excessive use of broad-spectrum agents frequently occur, particularly in high-risk surgical areas like neurosurgery and orthopedics [14,17]. The combined effects of these deviations result in a decline in preventive effectiveness, heightened selection for multidrug-resistant organisms, and intensified stewardship challenges.

In summary, although cefazolin is recognized as the gold standard for spinal prophylaxis, successful SAP necessitates a deeper understanding beyond mere adherence to guidelines. It requires the meticulous consideration of timing, dosing, surgical context, and patient risk. Strategies involving dual agents and alternative regimens should only be utilized in situations where there are definitive indications. Furthermore, it is essential for real-world practices to adhere to stewardship principles to mitigate the emergence of resistant pathogens.

### Global Variability and Misuse of Surgical Prophylaxis

The results from the 2022–2023 European Centre for Disease Prevention and Control (ECDC) point-prevalence survey (PPS), which includes 28 EU/EEA countries, highlight the extent of the problem of surgical prophylaxis [2]. In acute care hospitals, 14.9% of all antibiotics prescribed were attributed to SAP, similar to the percentage of antibiotics for the treatment of hospital infections (18%). It is concerning that 48% of all prophylactic regimens surpassed the advised 24 h duration, with numerous cases extending to 72 h or longer without appropriate clinical justification. The tendency for prolonged durations was especially notable in high-volume surgical centers, where preventative measures are frequently shaped by personal anecdotes or concerns about legal implications instead of established guidelines.

A significant issue is the misuse of broad-spectrum antibiotics in sterile spinal surgeries. Ceftriaxone, a third-generation cephalosporin noted for its ecological implications, has been one of the most commonly used agents for SAP across various European nations [2,3]. Ceftriaxone, despite its broad Gram-negative coverage, does not present any benefits over cefazolin in clean orthopedic or neurosurgical procedures. Additionally, it raises the risk of *Clostridioides difficile* infections and the selection of extended spectrum beta lactamase (ESBL)-producing organisms. Its extensive application in prophylaxis, particularly when lacking microbial justification, highlights a significant deficiency in the implementation of stewardship practices.

Neurosurgical departments, especially those engaged in intricate spinal procedures, face significant risks related to the misuse of SAP. Numerous investigations indicate that prolonged vancomycin prophylaxis, often alongside cefazolin and maintained postoperatively for as long as 72 h, is commonly given without formal testing for MRSA colonization [8,13,18]. Although these regimens aim to lower the risk of infection in revision fusions or multilevel constructs, the evidence supporting their use is not substantial and could potentially lead to new risks. 

## 3. Comparative Evidence on Vancomycin and Cefazolin in Spinal Surgery Prophylaxis

A significant amount of clinical evidence has investigated the comparative effectiveness and safety of vancomycin and cefazolin in preventing surgical site infections (SSIs) during spinal procedures. The studies encompass a diverse range of methodologies, such as randomized trials, retrospective cohorts, and prospective observational designs. The variations are evident not only in the study design, but also in the patient risk profiles, the extent of instrumentation, the methods of antibiotic delivery (IV versus topical versus combination), and the protocols for SSI surveillance. This section consolidates findings from thirteen significant studies, emphasizing methodological rigor, antimicrobial combinations, and microbiological outcomes, as outlined in Supplementary Table S1.

### 3.1. Individual Study Outcomes and Comparative Efficacy

Evidence supporting the use of vancomycin has primarily been derived from observational studies, especially in cases where topical or localized administration was implemented alongside standard intravenous prophylaxis. Park et al. [18] performed a retrospective cohort analysis involving 1074 patients who underwent spinal instrumentation. Their findings indicated that the use of both IV and topical vancomycin led to a reduction in SSI rates to 0.37%, in contrast to the 1.23% observed in the IV-only group. In a similar study, Gaviola et al. [19] conducted a retrospective cohort analysis involving 326 patients who underwent multilevel fusion. They observed a noteworthy reduction in infection rates, decreasing from 11% to 5.2% with the addition of vancomycin powder to IV cefazolin. Sweet et al. [20] conducted a retrospective cohort study involving 1732 thoracolumbar fusions, revealing that the use of adjunctive topical vancomycin significantly reduced the incidence of deep surgical site infections from 2.6% to 0.2%, with no indications of resistance observed. Data from further studies also corroborate these findings: Chotai et al. [21] conducted a longitudinal cohort study involving 2802 spinal surgeries and found a decrease in deep surgical site infections from 2.5% to 1.6% following the application of vancomycin (*p* = 0.02), with no resistant strains detected. Burak et al. [22] conducted a prospective comparative study, revealing that immersing pedicle screws in a vancomycin–cefazolin solution led to a reduction in infection rates from 15.6% to 8.7%, indicating a potential protective effect of localized delivery.

The evidence supporting cefazolin is predominantly derived from high-quality studies, particularly randomized trials. Herrington et al. [14], in their analysis of data from a randomized controlled trial with 535 spine surgery patients, observed that the SSI rates were significantly higher in the vancomycin group (3.5%) compared to the cefazolin group (1.4%), suggesting that impaired tissue penetration may limit the effectiveness of vancomycin. Nishant et al. [23] conducted a randomized study involving 90 patients assigned to receive cefazolin, cefuroxime, or ceftriaxone. The findings indicated comparable infection rates among all groups, leading to the recommendation of cefazolin due to its narrower spectrum and stewardship profile. Prospective data support the efficacy of cefazolin: Amelot et al. [24], in two prospective cohorts comprising 2250 non-instrumented spine surgeries, demonstrated a decrease in SSI from 4.9% to 1.7% with cefazolin prophylaxis (*p* < 0.0001). In a similar vein, Pomares et al. [11] conducted a three-cohort prospective study involving 132 high-risk patients, revealing that extended cefazolin–amikacin prophylaxis resulted in the lowest SSI rate (2.5%), thereby reinforcing the pivotal role of cefazolin in prophylactic measures.

Ambiguous results have been observed, as multiple extensive observational studies have not demonstrated a definitive benefit of one agent compared to another. Nguyen et al. [7] conducted a retrospective analysis of 859 neurosurgical patients, revealing no significant difference in SSI rates between cefazolin (2.2%) and vancomycin (4.1%). Lopez et al. [8], in a retrospective cohort of 3231 spinal fusion cases, found that dual cefazolin–vancomycin prophylaxis reduced SSI rates in revision surgeries by half (from 4% to 2%), indicating a potential advantage in specific high-risk situations rather than overall superiority. In a case–control study involving 4878 patients, Khanna et al. [25] found no increase in vancomycin-resistant organisms despite extensive topical use; however, they did observe an increase in Gram-negative infections. In contrast, Tafish et al. [26], through a retrospective cohort study involving 456 patients, observed no significant decrease in SSIs with the use of topical vancomycin (8.9% vs. 5.3%), raising questions about its effectiveness in routine-risk scenarios.

Collectively, these findings indicate that the observed advantage of vancomycin primarily arises from observational cohorts utilizing topical or adjunctive approaches, frequently in high-risk or multilevel procedures. In contrast, robust randomized and prospective evidence consistently endorses cefazolin as the standard agent, highlighting its advantages of a narrower spectrum, reduced toxicity, and enhanced alignment with stewardship principles. The ambiguous studies emphasize that the effectiveness of prophylaxis can be significantly influenced by the context, fluctuating with the complexity of the surgery, the prevalence of MRSA, and the local resistance patterns. In summary, the preponderance of controlled evidence supports the use of cefazolin as the primary choice for prophylaxis, while vancomycin is indicated as an additional option for specific high-risk groups.

### 3.2. Comparative Insights and Strategic Approaches

In terms of a monotherapy comparison, research conducted by Nguyen [7], Herrington [14], and Nishant [23] consistently showed that cefazolin performs comparably or even better than vancomycin. Vancomycin monotherapy, particularly in the absence of MRSA screening, seemed to be less effective in preventing SSIs and could potentially elevate the risk of shifts in Gram-negative flora [25]. 

In terms of dual-agent protocols, robust findings from Lopez [8], Park [18], and Sweet [20] substantiate the application of combination regimens in high-risk or revision surgeries. The findings from these studies indicate the lowest overall SSI rates, ranging from 0.2% to 0.37%, were especially noted with the concurrent application of topical treatments. The combination of systemic and localized antimicrobial activity could improve intraoperative protection while avoiding an increase in resistance rates. 

The outcomes of topical vancomycin were varied. Gaviola [19], Chotai [21], and Burak [22] demonstrated positive results with the application within the wound. Nonetheless, Tafish [26] observed no notable advantages in patients categorized as moderate-risk, while Khanna [25] expressed apprehensions regarding ecological transitions favoring Gram-negative organisms. The results indicate that the application of topical vancomycin should be approached selectively, rather than adopted universally. 

### 3.3. Heterogenicity Across Studies

The interpretation of comparative evidence is constrained by significant variability among the included studies. First, definitions of surgical site infection (SSI) were inconsistent, with some studies distinguishing between superficial and deep infections, while others applied broader composite endpoints. Second, follow-up duration ranged from 30 days to more than 12 months, affecting the detection of late-onset infections. Third, prophylactic regimens varied: cefazolin dosing ranged from 1 g to 3 g with inconsistent redosing intervals, while vancomycin was administered intravenously, topically, or in combination, often without standardized infusion timing. These methodological differences complicate the direct comparison of SSI rates and likely contribute to the divergent outcomes observed. Such variability underscores the need for uniform prophylaxis protocols and consistent reporting in future research.

### 3.4. Cost-Effectiveness Considerations

In addition to clinical outcomes, cost-effectiveness analyses have underscored the potential economic impact of vancomycin prophylaxis in spine surgery. Godil et al. demonstrated that the application of local vancomycin powder in posterior spinal fusion for trauma significantly reduced the incidence of SSIs compared with a 13% infection rate in control groups, resulting in estimated cost savings of approximately USD 438,000 per 100 procedures [27]. Similarly, Emohare et al. reported that intrawound vancomycin prevented all SSIs in a cohort of 96 patients, whereas 7 infections occurred among 207 controls, generating an additional cost burden of more than USD 570,000 [28]. The acquisition cost of vancomycin powder was minimal (USD 12 per patient), especially when contrasted with the mean infection-related cost exceeding USD 30,000 per patient, making the intervention highly cost-effective in their analysis. These findings indicate that, in selected high-risk groups, vancomycin prophylaxis may reduce morbidity and deliver substantial healthcare savings. Nonetheless, given conflicting clinical evidence and the absence of benefit in randomized controlled trials, such cost-effectiveness advantages should be carefully weighed against stewardship concerns and the potential for antimicrobial resistance.

### 3.5. Implications for Resistance and Stewardship

No significant emergence of vancomycin-resistant enterococci (VRE) was noted across the studies. Nonetheless, constraints in the duration of follow-up and the monitoring of microbiological factors impede the ability to draw definitive conclusions. It is important to highlight that Sweet [20], Khanna [25], and Chotai [21] found no significant emergence of VRE; however, it is worth noting that only Khanna [25] conducted systematic monitoring of resistance trends over a decade.

The potential for ecological imbalance continues to be a significant issue. Multiple investigations, especially the study by Khanna [25], have shown a significant transition toward Gram-negative organisms with the widespread use of vancomycin. This underscores the necessity for targeted, indication-based prophylaxis instead of blanket application.

Recent findings highlight that the consequences of vancomycin prophylaxis reach far beyond just resistance dynamics. Herrington et al. [14] found that the risk of surgical site infection was 2.5 times greater with vancomycin than with cefazolin, even when controlling for factors like obesity, revision surgery, and depression. This underscores that the selection of prophylactic measures affects both microbial ecology and immediate surgical results. A systematic review and meta-analysis conducted by Bakhsheshian et al. [29] highlighted significant variability among studies regarding vancomycin powder. Some cohorts indicated a decrease in superficial infections, whereas others reported no advantages, and even expressed concerns about the potential for resistance selection. In a significant advancement, Nascimento et al. [30] executed the inaugural double-blind randomized controlled trial in spinal arthrodesis, revealing that the use of intrawound vancomycin did not result in a reduction in SSI rates when compared to the placebo, thereby directly questioning established beliefs regarding its effectiveness. This study stands out as the most robust evidence regarding the use of intrawound vancomycin in spinal arthrodesis. This trial revealed no reduction in surgical site infection rates compared with the placebo, directly questioning the established practice of routinely applying intrawound vancomycin, which had primarily been supported by retrospective and observational studies. The absence of benefits in this high-quality trial highlights the limitations of relying on non-randomized evidence, where confounding factors and selective reporting can skew results. Accordingly, although adjunctive or topical vancomycin has demonstrated favorable trends in some retrospective studies, the Nascimento trial strongly suggests that such approaches should not be adopted universally and should instead be reserved for carefully selected high-risk cases until further randomized trials are available. In addition, Wang et al. [31] conducted a meta-analysis that synthesized the available evidence regarding the use of topical antibiotics in spine surgery. While some specific subgroups seemed to gain advantages, they determined that the general reliability of the evidence is still low and inadequate to support widespread, universal application.

Therefore, it is essential for hospitals to monitor and publicly disclose SAP compliance metrics within the context of comprehensive surgical quality dashboards. Moreover, it is crucial to formalize collaboration among surgeries and infectious disease services to establish stewardship audit loops that can effectively identify and rectify patterns of overuse or deviation. The absence of interdisciplinary consultation, especially in environments lacking regular engagement from infectious disease specialists or clinical pharmacologists, further detaches SAP decision making from modern stewardship principles and leads to ongoing inconsistencies in prophylaxis practices. Neurosurgical institutions should actively lead in aligning their prophylaxis protocols with the best available evidence, ensuring that the operating room serves as a focal point for stewardship rather than contributing to the resistance crisis.

### 3.6. Changes in Protocol and Their Effects in Real-World Scenarios

The analyzed studies underscore a significant concern: inconsistency in SAP protocols among different institutions, which is affected by local MRSA prevalence, surgeon preference, institutional procurement policies, and regulatory oversight. For instance, Amelot [24] and Pomares [11] implemented narrow-spectrum regimens with precise timing, whereas research by Lopez [8] and Park [18] preferred broader or dual regimens in more intricate scenarios. Burak’s unique screw-immersion approach exemplifies a novel application in local prophylaxis. 

The observed variations highlight the critical need for stewardship frameworks to align SAP utilization with clinical evidence. Focusing on screening, local antibiograms, and pharmacodynamic profiles is essential. In summary, cefazolin continues to be the main choice for prophylaxis in clean spinal surgery, due to its effective balance of efficacy, cost, and resistance profile. The application of vancomycin, whether administered systemically or topically, ought to be informed by discernible risk factors, including MRSA colonization or the presence of multi-level instrumented procedures. Utilizing dual-agent prophylaxis presents considerable advantages in high-risk scenarios; however, its widespread application poses microbiological and ecological challenges. Future guidelines ought to embody stratified prophylactic strategies that are customized to the specific risks associated with patients and procedures.

### 3.7. Clinical Recommendations for Practice

Drawing from the existing data, a number of actionable suggestions can be proposed (Table 1). Cefazolin alone should continue to be the standard prophylactic agent for the majority of clean spinal procedures, including primary fusions and non-instrumented surgeries, due to its reliable pharmacokinetics, effectiveness against *Staphylococcus aureus*, and favorable safety profile. Selective dual prophylaxis with cefazolin and vancomycin may be warranted in high-risk situations, including revision surgeries, multi-level instrumented procedures, or in facilities with a documented high prevalence of MRSA; however, routine application lacks support from controlled evidence. Vancomycin monotherapy is not recommended, except for patients with a confirmed β-lactam allergy or documented MRSA colonization; its delayed bone penetration, variable pharmacodynamics, and potential ecological consequences limit its effectiveness when used empirically. These recommendations reflect stewardship principles by emphasizing the importance of narrow-spectrum, timely, and short-duration prophylaxis, while reserving broader regimens for well-defined risk groups.

### 3.8. Study Limitations

This review has several important limitations. First, it is a narrative rather than a systematic review, and although a structured search strategy was applied, potential selection bias cannot be excluded. Second, no meta-analysis was performed because of substantial variability in study design, outcome definitions, follow-up duration, and prophylaxis regimens, which precluded reliable quantitative synthesis. Third, most of the evidence included was derived from retrospective or single-center studies, limiting the generalizability and increasing susceptibility to confounding. Finally, outcome reporting was inconsistent, with variable use of statistical testing and incomplete denominators, further constraining interpretation. These limitations highlight the need for rigorously designed multicenter randomized controlled trials to provide clearer insights into the comparative effectiveness of cefazolin and vancomycin in spinal surgery prophylaxis. In addition, the absence of a meta-analysis and the inconsistent reporting of denominators, confidence intervals, and effect sizes across studies limit the strength of statistical synthesis and constrain the reliability of direct comparisons.

## 4. Materials and Methods

**Figure 1:**
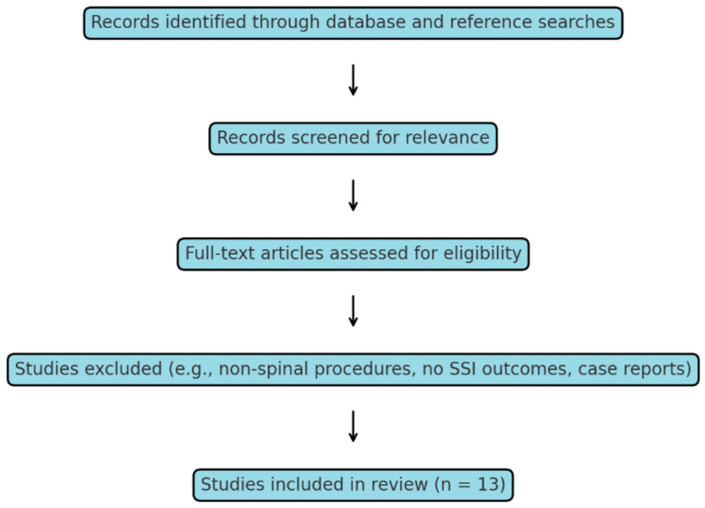
Simplified flow diagram of study selection for the narrative review. A total of 13 studies met the inclusion criteria and were included in the synthesis.

This narrative review aimed to assess the comparative effectiveness and safety of vancomycin compared to cefazolin in the context of surgical antibiotic prophylaxis for spinal procedures. A thorough examination of the literature was conducted through keyword-driven searches in PubMed and Scopus, complemented by manual reference screening. Combinations of terms such as “spine surgery”, “surgical site infection”, “prophylaxis”, “vancomycin”, “cefazolin”, “MRSA”, “VRE”, and “resistance” were included. The investigation encompassed the timeframe from January 2008 to August 2025, with the final search conducted on 3 August 2025, and was enhanced by a thorough manual review of reference lists. Studies that met the criteria were clinical investigations, including randomized controlled trials, cohort studies, or case–control designs, which assessed surgical antibiotic prophylaxis in spinal surgery. These studies specifically involved cefazolin and/or vancomycin, either individually or in combination, and reported outcomes related to surgical site infection (SSI) rates or microbiological findings. The criteria for exclusion included non-spinal procedures, case reports, conference abstracts, editorials, and studies that did not provide data on SSI outcomes. A total of 13 studies fulfilled these criteria and were incorporated for synthesis (Figure 1). The lack of a formal risk of bias or study quality assessment is due to the diverse designs, outcome definitions, and reporting methods present in the studies. This gap is a significant limitation and is clearly stated in the Section 3.8.

The findings from these studies were compiled into a comparative evidence table (Supplementary Table S1), detailing sample size, antibiotic protocols, SSI incidence, and significant conclusions. There was no formal quality assessment or meta-analysis performed because of the variability in methodologies.

To provide context for the findings, data on antimicrobial stewardship from the European Centre for Disease Prevention and Control (ECDC) were incorporated. The data served to demonstrate actual trends in SAP implementation and underscore the variability present among healthcare systems.

## 5. Conclusions

This review provides an overview of the current understanding of antibiotic prophylaxis in spinal surgery, emphasizing the impact of procedure type, operative duration, revision status, and patient- or institution-specific risk factors on infection outcomes. Data consistently indicate that cefazolin is the standard prophylactic agent, whereas vancomycin should be reserved for patients with MRSA colonization, β-lactam allergy, or carefully selected high-risk revisions. Routine application of vancomycin lacks justification and may alter microbial patterns, favoring Gram-negative and opportunistic organisms.

In various surgical disciplines, practice is often shaped by institutional routines and surgeon preference, which are not always grounded in strong evidence. Minimizing surgical site infections requires strict compliance with established prophylaxis protocols, combined with a stewardship-oriented approach that balances clinical benefit against the risk of resistance. Exploring all potential strategies to improve prevention through multicenter randomized trials, standardized definitions, and economic evaluation is essential to advancing patient outcomes and preserving antimicrobial effectiveness.

## Figures and Tables

**Table 1:** Practical recommendations for surgical prophylaxis in spinal surgery.

Patient Group/Surgical Context	Recommended Prophylaxis	Notes/Rationale
Clean spinal procedures (primary, non-instrumented)	Cefazolin alone (standard dosing, timely redosing)	Effective against *S. aureus*; favorable pharmacokinetics; strong evidence base.
Instrumented primary procedures (no MRSA risk factors)	Cefazolin alone	Remains adequate; vancomycin not indicated without colonization or allergy.
Revision or multi-level procedures in MRSA-endemic centers	Cefazolin + vancomycin (dual prophylaxis)	Consider only in high-risk cases; routine use not supported by controlled data.
Patients with confirmed MRSA colonization	Vancomycin ± cefazolin (depending on risk)	Vancomycin justified; may combine with cefazolin in revision/high-risk cases.
Patients with β-lactam allergy	Vancomycin monotherapy	Reserved for true allergy; empirical use not recommended.

## Data Availability

No new data were created or analyzed in this study. Data sharing is not applicable to this article.

## Supplementary Materials

The following supporting information can be downloaded athttps://www.mdpi.com/article/10.3390/antibiotics14100996/s1, Table S1: Summary of Key Clinical Studies Evaluating Prophylactic Use of Vancomycin and Cefazolin in Spine Surgery.

## Abbreviations

The following abbreviations are used in this manuscript:

AMR	Antimicrobial Resistance
CDC	Centers for Disease Control and Prevention
CoNS	Coagulase-Negative Staphylococci
ECDC	European Centre for Disease Prevention and Control
ESBL	Extended-Spectrum Beta-Lactamase
HAIs	Healthcare-Associated Infections
MDROs	Multidrug-Resistant Organisms
MIC	Minimum Inhibitory Concentration
MRSA	Methicillin-Resistant Staphylococcus aureus
MSSA	Methicillin-Sensitive Staphylococcus aureus
NASS	North American Spine Society
SAP	Surgical Antibiotic Prophylaxis
SSI	Surgical Site Infection
VRE	Vancomycin-Resistant Enterococci
