# Supplementary material for: Does Vancomycin as the First-Choice Therapy for Antibiotic Prophylaxis Increase the Risk of Surgical Site Infections Following Spine Surgery?
Source: Antibiotics (Basel). 2025 Oct 5;14(10):996. doi: 10.3390/antibiotics14100996 (PMC12561592; doi:10.3390/antibiotics14100996)
Supplement: Supplementary file 1 [file antibiotics-14-00996-s001.zip › antibiotics-3869496-supplementary.pdf]

Group by administration type	Study	Design	n (Total/Groups)	Antibiotic Regimens	SSI % (n/den)	p-value / Key Outcome
**IV Only**	Nguyen et al.	Retrospective cohort	859 (Cef 664 / Vanc 195)	IV cefazolin vs IV vancomycin	2.2% (14/664) vs 4.1% (8/195)	p=0.14, No significant difference
**IV Only**	Herrington et al.	Retrospective analysis of RCT	535 (Cef 223 / Vanc 312)	IV cefazolin vs IV vancomycin	1.8% (4/223) vs 3.5% (11/312)	p=0.03, Higher SSI with vancomycin
**IV Only**	Lopez et al.	Retrospective cohort	3231 (Cef only 1426 / Cef+Vanc 1805)	IV cefazolin vs IV cefazolin + IV vancomycin	4.0% (57/1426) vs 2.0% (44/1805)	p<0.001, Combination reduced SSI and revisions
**IV Only**	Amelot et al.	Prospective	2250 (No-AB 1031 / Cef 1219)	No prophylaxis vs IV cefazolin	4.9% (51/1031) vs 1.7% (21/1219)	p<0.0001, Single-dose cefazolin effective
**IV Only**	Pomares et al.	Prospective 3-cohort	132 (46/46/40)	Cefazolin vs Cefazolin+Amikacin (24h / 72h)	23.9% vs 8.7% vs 2.5%	Prolonged double therapy decreased SSI
**IV Only**	Nishant et al.	Prospective RCT	90 (33/30/27)	Cefazolin vs Cefuroxime (1g vs 1.5g)	0% vs 1/30 vs 1/27	No difference, single pre-op dose adequate
**Topical**	Gaviola et al.	Retrospective cohort	326 (IV Cef 210 / IV Cef + Topical Vanc 116)	IV cefazolin vs IV cefazolin + topical vancomycin	11% (23/210) vs 5.2% (6/116)	Topical vancomycin lowered SSI in multilevel fusion
**Topical**	Sweet et al.	Retrospective cohort	1732 (812 / 911)	IV cefazolin vs IV cefazolin + topical vancomycin	2.6% (21/812) vs 0.2% (2/911)	p<0.001, Marked reduction in deep SSI
**Topical**	Khanna et al.	Case-control	4878 (~2358 / ~2520)	Topical vancomycin vs none	1.4% (33/2358) vs 0.8% (20/2520)	No increase in VRE; slight increase in gram-negative infections
**Topical**	Chotai et al.	Prospective longitudinal	2802 (1215 / 1587)	Intrawound vancomycin vs none	1.6% (19/1215) vs 2.5% (40/1587)	p=0.02, Lower SSI, no resistance
**Topical**	Tafish et al.	Retrospective cohort	456 (81 / 375)	Topical vancomycin vs none	9.9% (8/81) vs 5.3% (20/375)	No clear benefit, further studies needed
**Combined/ Other**	Park et al.	Retrospective cohort	1966 (1074 / 892)	IV standard + IV & topical vancomycin vs IV only	0.37% (4/1074) vs 1.23% (11/892)	p<0.05, Lower infection, no resistance
**Combined/ Other**	Burak et al.	Prospective comparative	239 (104 / 135)	Pedicle screw soak (vancomycin + cefazolin) vs none	8.7% (9/104) vs 15.6% (21/135)	p<0.05, Significant reduction
